# Consistent oviposition preferences of the Duke of Burgundy butterfly over 14 years on a chalk grassland reserve in Bedfordshire, UK

**DOI:** 10.1007/s10841-021-00327-6

**Published:** 2021-06-09

**Authors:** M. P. Hayes, E. Ashe-Jepson, G. E. Hitchcock, R. I. Knock, C. B. H. Lucas, A. J. Bladon, E. C. Turner

**Affiliations:** 1 Department of Zoology, University Museum of Zoology, Downing Street, Cambridge, CB2 3EJ UK; 2 Wildlife Trust for Bedfordshire, Cambridgeshire and Northamptonshire, Unit 2, St Johns Barn, Griffin Farm, Conger Lane, Toddington, Bedfordshire, LU5 6BT UK

**Keywords:** Butterfly, Calcareous grassland, Climate change, Habitat management, *Hamearis lucina*, Oviposition

## Abstract

**Abstract:**

The Duke of Burgundy butterfly (*Hamearis lucina*) is known to have specific habitat requirements for its larval foodplants. However, no studies have yet investigated whether these preferences vary over time or in relation to climate, and there is a paucity of data on whether management on reserves can replicate preferred conditions. Here, we build upon existing research to confirm which characteristics Duke of Burgundy prefer for their larval foodplants, whether preferences remain consistent across years, and whether conservation management on reserves can replicate these conditions. Fieldwork was carried out at Totternhoe Quarry Reserve, a chalk grassland site in Bedfordshire, UK. Confirming previous research, we found that large *Primula* plants in dense patches were chosen for oviposition, but that once chosen there was no preference to lay eggs on a plant’s largest leaf. Chosen foodplants were also more sheltered and in closer proximity to scrub than their controls. However, at a finer scale, we found little evidence for any preference based on differences in microclimate, or vegetation height immediately surrounding the plants. This suggests features that alter microclimatic conditions at a larger scale are relatively more important for determining the suitability of oviposition sites. Nearly all preferences remained consistent over time and did not vary between years. Management of scrub on the reserve was able to reproduce some preferred habitat features (high plant density), but not others (large plant size).

**Implications for insect conservation:**

The consistency of findings across years, despite inter-annual variation in temperature, rainfall and number of adults, indicates that the Duke of Burgundy is conservative in its foodplant choice, highlighting its need for specific habitat management. Targeted management for foodplants could form part of a tractable set of tools to support Duke of Burgundy numbers on reserves, but a careful balance is needed to avoid scrub clearance leaving plants in sub-optimal conditions.

## Introduction

The choice of oviposition location by adult female butterflies can be subject to strong selection pressure, as their relatively immobile larvae rely almost completely on female site selection to determine their chances of survival (Chew 1977; García-Barros and Fartmann 2009; Beyer and Schultz 2010) and butterflies commonly have distributions closely linked to that of their larval foodplants (Dennis et al. 2004). However, foodplant abundance is only one of many factors affecting butterfly range, and foodplants are often far more widespread than the species they support (Dennis and Shreeve 1991; Dennis et al. 2005; Turner et al. 2009; Hayes et al. 2018). Indeed, many butterfly populations have become extinct in locations where their foodplants are still abundant (Butterflies Under Threat Team 1986). This may be linked to fine scale habitat differences associated with foodplants, which can reduce larval survival or the chance that butterflies oviposit (Elmes and Wardlaw 1982a, 1982b). Factors found to determine foodplant suitability for butterfly oviposition include, but are not limited to: plant size, accessibility (Anthes et al. 2008) and phenology (Leon-Cortes et al. 2004); surrounding vegetation structure, degree of shelter and associated microclimate (Fartmann 2006; Loffler et al. 2013); aspect, slope (Turner et al. 2009); and foodplant chemical composition (Awmack and Leather 2002; Kurze et al. 2018). To conserve threatened butterfly species, a knowledge of factors determining the suitability of oviposition locations may be key to tailoring management regimes to produce foodplants that encourage female oviposition and support larval growth and survival.

The Duke of Burgundy butterfly (*Hamearis lucina*) is one such species that has extremely specific larval habitat requirements (Sparks et al. 1994; Fartmann 2006; Anthes et al. 2008; Turner et al. 2009; Goodenough and Sharp 2016). Its larvae are narrowly oligophagous, and eggs are laid only on plants of the genus *Primula*, with variation in plant quality rendering a large proportion unsuitable for oviposition (Nakonieczny and Kedziorski 2005; Anthes et al. 2008; Hayes et al. 2018). Indeed, female Duke of Burgundy often oviposit on plants that already have conspecific eggs present on them, despite other unoccupied *Primula* plants being present nearby. On one occasion, Kirtley (1997) found a single plant containing 45 eggs from several different females, with habitat factors apparently outweighing the costs of increased larval competition (Kirtley 1995). Similarly, in 2006, working on the same chalk grassland reserve as this study (Totternhoe Quarry Reserve, Bedfordshire, UK), Turner et al. (2009) found that only 2.4% (87/3686) of searched *Primula* plants had Duke of Burgundy eggs present. These plants were located by surveying areas known to support Duke of Burgundy on the reserve and plants that were used for oviposition contained on average 3.1 eggs, but some contained as many as ten. A second data set was also recorded at Totternhoe in 2006, where every *Primula* plant on the reserve was located and the whole site was searched for larval feeding damage. These results were mapped by Hayes et al. (2018) who found that only 0.95% (84/8819) of the total number of plants showed larval damage. Taken together, these results indicate that a very small percentage of *Primula* plants have eggs laid on them and that an even smaller percentage support the prolonged survival of Duke of Burgundy larvae.

The vast majority of Duke of Burgundy populations in the UK now occupy scrubby calcareous grasslands (Bourn and Warren 1998; Oates 2000; Jones et al. 2012). Historically, the species was more frequently encountered in woodland clearings (Newman 1871), but populations are thought to have been displaced from these sites due to the widespread decline of traditional coppicing. This resulted in open woodlands closing up and foodplants being shaded out (Sparks et al. 1994; Oates 2000; Fartmann 2006; Noake et al. 2008). On grassland sites, female Duke of Burgundy butterflies tend to lay eggs on larger plants in denser patches, associated with either nearby scrub or taller surrounding vegetation, providing *Primula* with a shaded root system (Oates 2000; Turner et al. 2009). Together, this is thought to provide a large food source and reduce the risk of desiccation, increasing resources for larvae throughout their development (Sparks et al. 1994; Fartmann 2006; Anthes et al. 2008; Turner et al. 2009). This is supported by Goldenberg (2004), who found that plants chosen for oviposition were less water stressed than controls. Given its recent shift out of sheltered woodland habitats and its apparent reliance on certain water conditions, projected regional warming (Intergovernmental Panel on Climate Change 2014) and increased likelihood of extreme weather events such as drought could pose serious threats to the Duke of Burgundy (Fartmann 2006; Turner et al. 2009). Therefore, it is important to quantify how flexible the butterfly’s habitat preferences are across years and in different weather conditions, to assess its vulnerability to future climate change.

Management for the Duke of Burgundy on reserves often involves manual scrub clearance or grazing to stop early successional *Primula* plants being swamped by encroaching woody vegetation (Bourn and Warren 1998). Brys et al. (2004) found that clearance of vegetation can be used to maintain high numbers of *Primula* and promote high seed output for future years, but that management must occur at the right time of year. Mowing or grazing in the autumn, after the main period of *Primula* growth, had the best results (Brys et al. 2004). Goodenough and Sharp (2016) also found that grazing in the autumn increased *Primula* abundance, but that if this was undertaken at the wrong time of year or at the wrong intensity it could also cause plants to grow in sub-optimal forms for the Duke of Burgundy. Moderate grazing pressure during the autumn was found to have the best results and to support the highest number of large and succulent plants. They also found that grazing should be spread across a whole site instead of being confined to smaller sections on rotation, so that grazing intensity is not too high in any area (Goodenough and Sharp 2016). Fartmann (2006) also investigated the effects of different grazing regimes, but this time directly investigated their impact on the occurrence and density of Duke of Burgundy populations. The study found that traditional rough cattle grazing, taking place from late summer onwards, supported the highest densities of eggs and adult butterflies. Fartmann (2006) suggested that this could be due to the way cattle feed. By pulling and cropping vegetation with their tongue, cattle produce a diverse sward height, which can promote *Primula* plant growth, whilst also providing shelter for oviposition. Timing intervention until after the larvae pupate, from late summer onwards, is also likely to minimise disturbance to the butterfly (Butterflies Under Threat Team 1986; Bourn and Warren 1998).

Here we build upon and verify the work of previous studies, to investigate fine scale ovipositional preferences of the Duke of Burgundy at Totternhoe Quarry, a chalk grassland reserve in Bedfordshire, UK. By comparing physical habitat attributes and direct measures of microclimate in close proximity to larval-damaged foodplants with those of undamaged controls, we examine the specific habitat requirements of this life stage. For the first time, we compare habitat and microclimatic preferences collected using comparable methods in five different years over a 14-year period (2006, 2007 (results previously published in Turner et al. (2009)), 2009, 2016 and 2020) to assess whether any preferences vary over time. Finally, we compare *Primula* plant characteristics in paired scrub cut and unmanaged areas at Totternhoe Quarry, to assess the effects of management and whether scrub cutting results in plants with features selected by Duke of Burgundy for oviposition.

## Methods

### Study site

All fieldwork was carried out at Totternhoe Quarry Reserve in Bedfordshire (latitude = 51:89 N, longitude = 00:57 W), an area of unimproved chalk grassland owned and managed by the Wildlife Trust for Bedfordshire, Cambridgeshire and Northamptonshire (BCN Wildlife Trust). At 13.6 hectares, the site is small, but supports several rare species of plants and invertebrates, including a good population of Duke of Burgundy butterflies, which have been studied for over 10 years. Additional land has gradually been acquired by the BCN Wildlife Trust to expand the site and link it to other protected areas, forming a larger Totternhoe reserve network (BCN Wildlife Trust, Totternhoe 2020). For more details, see Turner et al. (2009), Hayes et al. (2018) and Hayes et al. (2019).

We obtained regional climatic data from the MET Office to quantify differences in rainfall and temperature between recording years. Data on daily rainfall, mean, daily maximum and daily minimum temperature were acquired from the Woburn weather station (latitude = 52:01 N, longitude = 00:59 W), just over ten miles from Totternhoe Quarry Reserve, for April, May and June (when females were selecting sites for oviposition) in each recording year (Met Office 2006). The first date that adult Duke of Burgundy butterflies were observed, and the date peak numbers of adults were counted at Totternhoe Quarry, were also obtained for each recording year from the Biodiversity Recording and Monitoring Centre for Bedfordshire and Luton (BRMC). Data were gathered from a standardised transect through representative habitats on the reserve, which was walked weekly at Totternhoe throughout the flight season of the Duke of Burgundy, as part of the Butterfly Monitoring Scheme (UKBMS, Methods 2020). Regional climatic variables showed some interannual variability across the 14 years of this study. Mean temperature ranged from 11.8 to 12.7 °C, with mean daily maximum and mean daily minimum temperatures ranging from 16.4 to 18.9 °C and 6.5 to 7.7 °C respectively. Rainfall ranged from an average of 0.9–2.7 mm per day (Table 1). The first date adult Duke of Burgundy butterflies were observed on site and the date peak numbers were recorded also varied across years, ranging from April 21st–May 12th and April 25th–May 23rd respectively, with the peak numbers themselves ranging from 5 to 14 adults observed (Table 1).

**Table 1:** Regional climate data obtained from the MET Office Woburn weather station, less than ten miles from our study site at Totternhoe Quarry

Aspect of climate/Duke of Burgundy population data	2006	2007	2009	2016	2020
Mean daily minimum temperature (°C) (± SE)	7.4 (± 0.4)	7.7 (± 0.4)	7.3 (± 0.3)	7.1 (± 0.5)	6.5 (± 0.4)
Mean temperature (°C) (± SE)	12.4 (± 0.4)	12.7 (± 0.3)	12.6 (± 0.3)	11.8 (± 0.4)	12.7 (± 0.4)
Mean daily maximum temperature (°C) (± SE)	17.4 (± 0.5)	17.7 (± 0.3)	17.8 (± 0.4)	16.4 (± 0.4)	18.9 (± 0.5)
Mean daily rainfall mm (± SE)	1.4 (± 0.3)	2.2 (± 0.5)	0.9 (± 0.3)	2.7 (± 0.8)	1.3 (± 0.4)
First Duke observation/emergence date	11/05/2006	01/05/2007	29/04/2009	12/05/2016	21/04/2020^a^
Date of peak count	18/05/2006	22/05/2007	29/04/2009	23/05/2016	25/04/2020^a^
Number of adults observed during peak count	14	7	5	10	11^a^

Data on mean, daily maximum, daily minimum temperature and daily rainfall are displayed (± one standard error) for the months of April, May and June (the flight period of the Duke of Burgundy), for each of the five years of this study. The first date adult Duke of Burgundy butterflies were observed, and the date peak numbers of adults were counted at Totternhoe Quarry were also obtained for each year of the study from the Biodiversity Recording and Monitoring Centre for Bedfordshire and Luton (BRMC)

^a^The first covid-19 lockdown in England in March of 2020 coincided with the Duke of Burgundy flight season, which reduced the possible number of survey days

### Foodplant habitat attributes

From June to August 2016, the entire Totternhoe Quarry Reserve was systematically surveyed for *Primula* foodplants that showed the distinctive peppering damage pattern produced by Duke of Burgundy larvae (Oates 2000; Turner et al. 2009). In 2020 the survey was repeated over the entire reserve but slightly later in the year (August only), owing to the restrictions of the COVID pandemic. The later survey date and associated growth of surrounding vegetation may, therefore, mean that damaged plants were less likely to be located in 2020. Work was carried out with the help of Wildlife Trust staff, volunteers and a number of university students. Throughout the surveys, we were conservative as to which plants were recorded, choosing only those which showed unambiguous Duke of Burgundy larval damage. Our criteria for inclusion followed the methods of Hayes et al. (2018) and were as follows: 1) plant damage had to be concentrated in the centre of the leaves, with the majority of veins, midribs and leaf edges remaining intact; 2) plants had to display a progression of increasing hole sizes, produced as larvae grew and moved away from the leaf where eggs were oviposited. When damage was found, measurements of the plant and its surrounding habitat attributes were recorded, following Turner et al. (2009). When several plants were found in a single area, they were recorded as separate individual plants when leaves did not overlap. Measurements included: the number of other *Primula* plants within a 30 cm radius of the focal larval damaged plant (again only counting separate surrounding plants if leaves did not overlap), the length of the longest leaf, and the length of the leaf with the smallest larval damage holes (termed the “egg leaf” and likely to be the leaf on which the eggs were laid, as damage holes increase with larval size). Average vegetation height surrounding the plant was also measured by gently resting an A4 clipboard on the surrounding vegetation and measuring the height of the clipboard from the ground. This method is advocated by Stewart et al. (2001) who refer to it as ‘the direct method’ for measuring sward height. We also recorded the distance of the plant to the base of the nearest woody vegetation. Finally, we assessed the overall degree of shelter within close proximity of the plant using a five-point scale, with a shelter value of one signifying that all cardinal directions (north, east, south and west) were open and free from a barrier at one metre height for at least five metres, but a shelter value of five signifying that all four points of the compass were obstructed within this distance. A barrier could consist of any solid obstruction and included woody vegetation or steep banks (Turner et al. 2009; Hayes et al. 2018). Finally, in 2016, a photograph of each plant was taken from above, alongside a numbered scale bar, allowing us to calculate plant size (surface area) using ImageJ visual processing software (ImageJ 2020) and a Wacom Intuos pro (PTH-851) drawing tablet. In 2020, approximate plant size (surface area) was calculated using the equation (Maximum length X Maximum width)/2, which equates the size of a diamond and follows the methods of Turner et al. (2009).

To compare measurements that vary at a small-scale (plant size, plant density, leaf length, vegetation height and distance to scrub), we selected a paired control plant in the immediate area of each larval damaged plant and repeated all measurements in a comparable way. Control plants were chosen by selecting the undamaged *Primula* plant lying closest to one metre from the focal damaged foodplant in the direction north, east, south or west, with the direction being rotated sequentially to avoid bias.

For habitat features that are also or only likely to vary at larger scales, beyond a distance of one metre from focal foodplants (degree of shelter and a repeat measure of distance to scrub that assessed this preference at a larger scale), we selected unpaired control plants for comparisons. Using ArcMap GIS software (ArcMap 2020) and a 2016 map of the reserve (see Hayes et al. 2018), we generated two sets of random points across Totternhoe Quarry to match the number of larval damaged plants we had recorded in 2016 and 2020. In April 2017 and September 2020 respectively, we located the *Primula* plant closest to each of these points and used them as controls to measure shelter and distance to scrub. As neither degree of shelter or distance to scrub are likely to vary with time of year, the later timing of these surveys is unlikely to influence results.

### Foodplant microclimate

In August 2009, Tinytag data loggers (Gemini Tinytag TGP-4510 Data Loggers) were placed at the base of 15 pairs of larval damaged plants and their controls (located within one metre of the larval damaged plants using the same methods as above) to compare temperature regimes, with readings being taken every hour over a two-day period. For a subset of six plants and their controls, relative humidity was also recorded. It should be noted that the microclimatic readings from August 2009 were likely taken after Duke of Burgundy larvae pupated. However, comparing foodplants selected for oviposition to controls still gives useful data on their relative differences in microclimate, even if data were collected later in the season than larvae were active. A similar, longer-term study was carried out from June 20th to July 19th 2016, using the first fourteen larval damaged foodplants and controls located during surveys. These plants were spread across different habitat types and reflect the variety of conditions available on the reserve. This time, iButton data loggers (DS1921G-F5 thermochrons) were placed at the base of the plants and temperature was recorded every three hours throughout the entire recording period.

### Cross year comparisons

To assess the consistency of foodplant preferences over time, we compared data on physical habitat attributes collected in 2016 and 2020 to data collected using comparable methods at the same site in 2006 and 2007 (Turner et al. 2009). However, in 2006 and 2007 foodplants were found by searching for eggs in known Duke of Burgundy hotspots, instead of searching for larval damaged plants across the whole reserve, as was done in 2016 and 2020. Although not all eggs will survive and develop into larvae that produce feeding damage, both methods of surveying allow plants used by Duke of Burgundy for oviposition to be located and analysed. Control plants were selected in the same way across all years, unless specifically stated otherwise. To avoid confusion we use the following terms throughout the rest of the paper: ‘foodplants’ when referring to plants selected for oviposition, which have been recorded by searching for eggs or larval damage; ‘control plants’ when referring to undamaged plants specifically selected as controls, and ‘*Primula* plants’ when referring to plants in general.

Comparable data sets for all four years existed for foodplant size, length of longest leaf, surrounding vegetation height and density of surrounding *Primula* plants. However, in our study we measured the number of other *Primula* plants within 30 cm radius of a focal plant, whereas Turner et al. (2009) measured this within a 50 cm radius. We therefore calculated differences in density between foodplants and their controls for both studies and compared this parameter between years to account for this methodological difference.

Other factors were only collected across three years of study. In 2006, 2016 and 2020 the length of the egg leaf was recorded and in 2007, 2016 and 2020 the distance to scrub was measured for foodplants and their controls. However, in 2007 distance to scrub was only measured using paired control plants lying within one metre of foodplants, not for unpaired controls across the reserve. Therefore, to allow comparison across years, measures of distance to scrub in 2016 and 2020 were also taken from paired controls lying within one metre of foodplants. All measures of temperature (mean temperature, variance, mean maximum and mean minimum daily temperatures) were available for comparison between 2009 and 2016 only.

### Scrub clearance and *Primula* regrowth

In June 2017, we located areas that had been actively cleared of scrub over the last five years at Totternhoe Quarry, as part of ongoing conservation management. Of 26 available areas with a size of approximately 50 m^2^ or more, ten had unmanaged dense scrub lying on a similar slope and aspect adjacent to them. These were selected as paired managed and unmanaged controls, in which *Primula* plant abundance and size were surveyed. We established three separate plots in both cleared and control locations from which to take measurements, by throwing up a one metre diameter hoop at random within each area. The number of *Primula* plants within each plot was then recorded and up to three *Primula* plants from each plot were selected for further measurements. If more than three plants were present in a plot, the three *Primula* plants closest to the edge, centre and 50 cm from the edge of each hoop were selected. The number of other *Primula* plants within 30 cm of each focal plant was counted and a photograph of each focal plant was taken with a scale. The size of each focal plant was measured from this image, using ImageJ software as detailed above. The time since scrub clearance in each of the managed areas was also recorded. However, we found that 90% were cleared within the preceding 18 months, so it was not possible to assess the long-term impact of time since scrub clearance on *Primula* plants.

### Analysis

We used R version 4.0.0, running the packages ‘plyr’, ‘dplyr’, ‘plotrix’, ‘dunn.test’, ‘ggplot2’ and ‘ggpubr’ for all analyses and generating figures.

### Foodplant habitat attributes

Because data were not normally distributed, we used non-parametric tests to compare habitat attributes of larval damaged foodplants with their undamaged controls. Data from 2016 and 2020 were pooled and Paired Wilcoxon tests were used to compare the size of the plants, number of other *Primula* plants within 30 cm, longest leaf length and surrounding vegetation height; with paired and unpaired Wilcoxon tests comparing the distance of the plants to scrub at small and large scales respectively. To assess differences in shelter, we used a Chi square goodness-of-fit test to compare the observed shelter values of larval damaged foodplants to randomly distributed controls. A further paired Wilcoxon test was used to compare the length of the longest leaf to the length of the egg leaf across all larval damaged foodplants.

### Foodplant microclimate

For 2009 and 2016, we calculated the mean temperature, variance, mean maximum and mean minimum daily temperatures for each plant over the two day and one month recording periods, for each year respectively. For 2009, we repeated all calculations for relative humidity levels. We used paired Wilcoxon tests to compare all microclimatic variables experienced by larval damaged foodplants and their controls in 2009 and 2016 separately.

### Cross year comparisons

Differences in physical habitat attributes between foodplants selected for oviposition and their paired controls were calculated for 2006, 2007, 2016 and 2020 separately, by subtracting control plant from foodplant attributes, and then compared across all years with available data. For all analyses comparing factors across multiple years, we used Kruskal–Wallis tests followed by Dunn’s post hoc tests with Bonferroni correction if significant differences were detected.

The differences between larval damaged foodplants and their controls (attribute value for control plant subtracted from foodplant) were also compared between 2009 and 2016 for all temperature variables (mean temperature, variance, mean maximum and mean minimum daily temperatures) using unpaired Wilcoxon tests.

### Scrub clearance and *Primula* regrowth

For each managed and control area, we calculated the mean number of *Primula* plants within each plot, the mean size of focal plants, and the mean number of other *Primula* plants within 30 cm. We used paired Wilcoxon tests to compare these values between managed and control locations, with differences in *Primula* size and number of surrounding plants within 30 cm only being analysed if both paired areas contained at least one plant.

To test whether *Primula* plants in managed areas were significantly smaller or were found at a lower density than foodplants chosen for oviposition, we compared size and number of plants around focal *Primula* plants in managed areas to the same characteristics for larval damaged foodplants found in 2016 using one-sided unpaired Wilcoxon tests.

## Results

### Foodplant habitat attributes

**Fig. 1:**
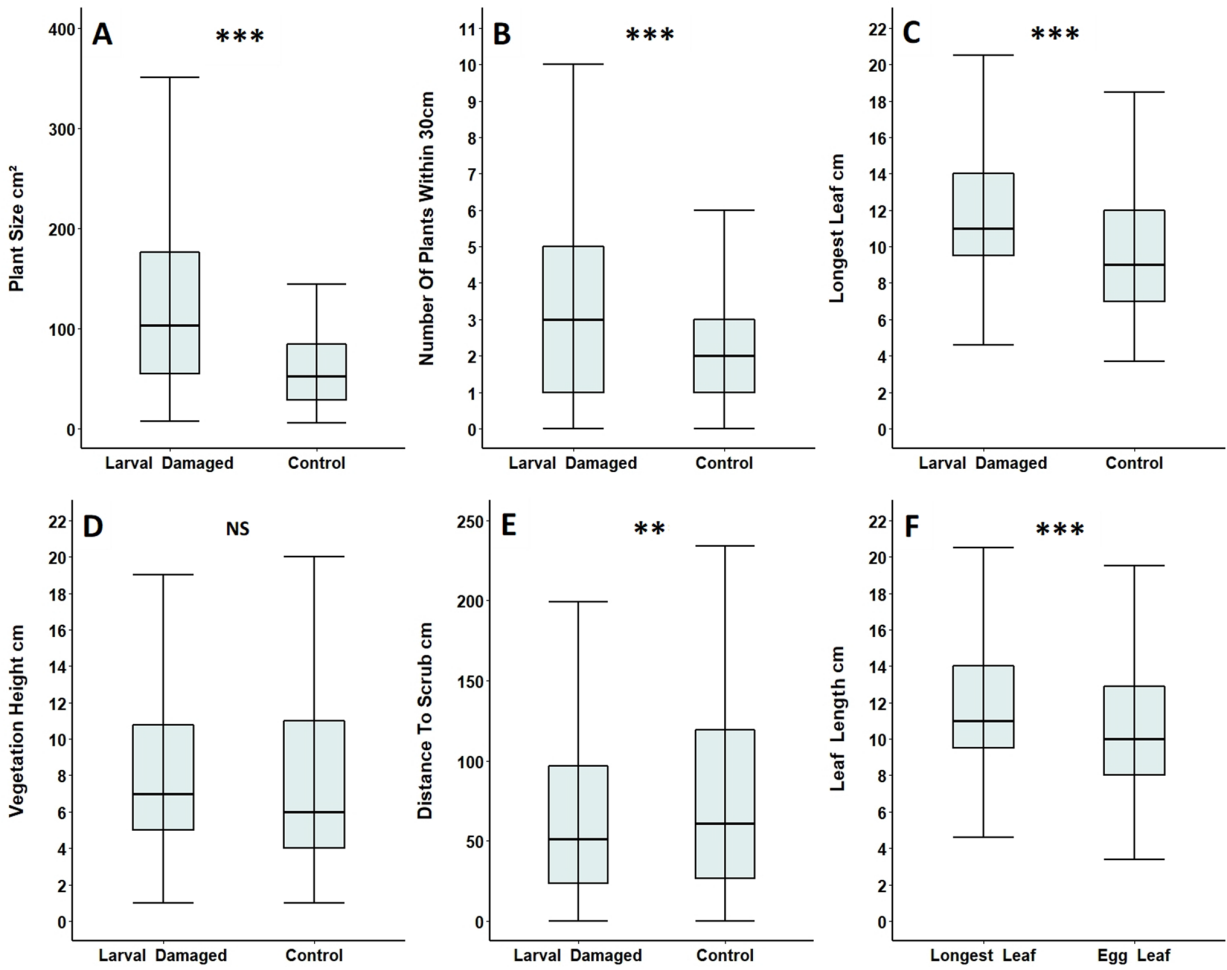
**A** Plant size, **B** number of other *Primula* plants within 30 cm, **C** longest leaf length, **D** surrounding vegetation height and **E** distance to scrub of larval damaged foodplants, compared to undamaged control plants, at Totternhoe Quarry Reserve in 2016 and 2020. For foodplants, the length of the ‘longest leaf’ was also compared to the length of the ‘egg leaf’ used for oviposition (**F**). All paired control plants were within one metre of the larval damaged foodplant. Box and whisker plots show median values, with boxes representing the interquartile range and whiskers extending to the largest value no more than 1.5 × the interquartile range. For ease of readability, outlying data points beyond this range are not displayed. **p < 0.01, ***p < 0.001, NS = no significant difference

**Fig. 2:**
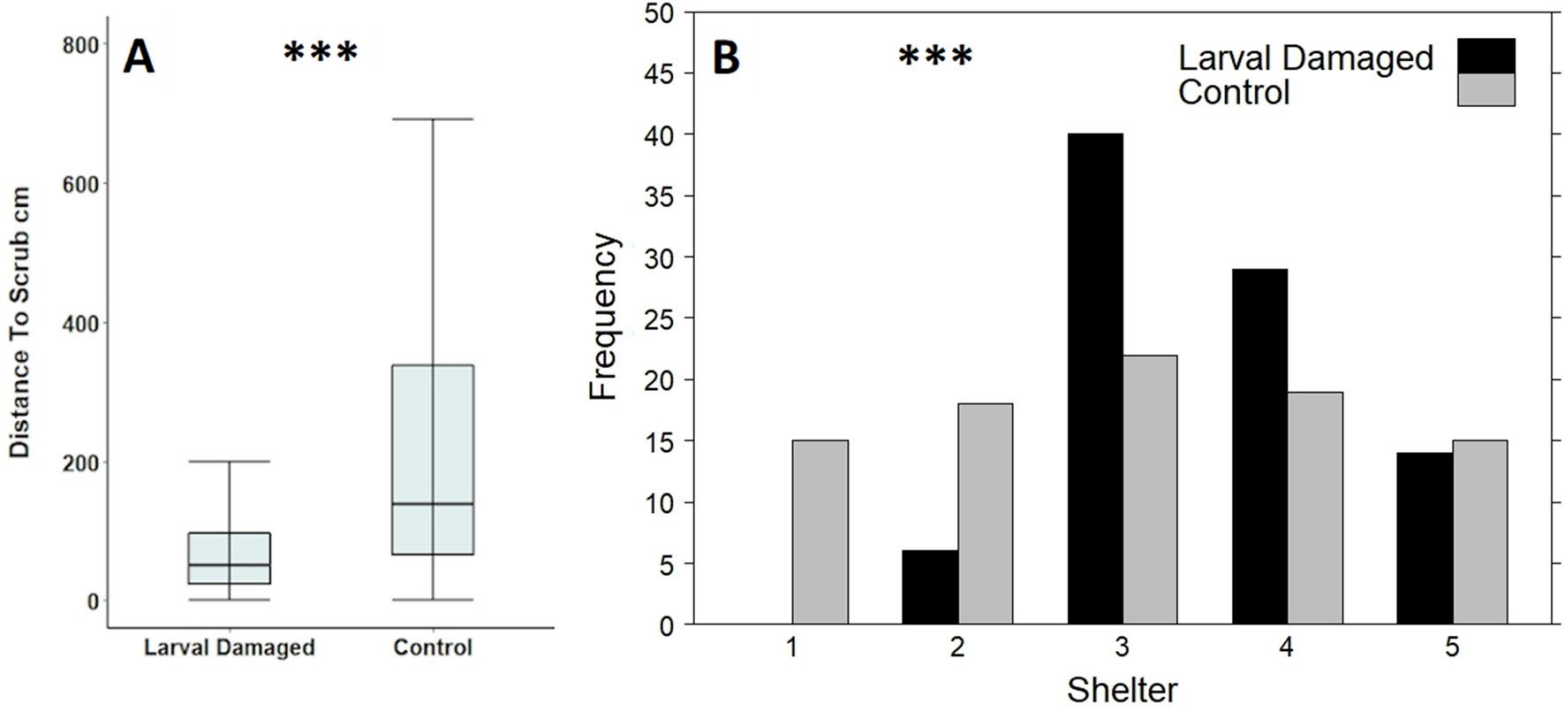
**A** Distance to scrub and **B** observed shelter values of larval damaged foodplants compared to that of unpaired randomly distributed control plants at Totternhoe Quarry Reserve in 2016 and 2020. A shelter value of one indicates that there was no barrier within five metres of the plant in all four cardinal directions (north, east, south and west), while a value of five indicates that a barrier was present in all four directions. Box and whisker plots show median values, with boxes representing the interquartile range and whiskers extending to the largest value no more than 1.5 × the interquartile range. For ease of readability, outlying data points beyond this range are not displayed. ***p < 0.001

In total, 110 *Primula* foodplants with Duke of Burgundy larval damage were located at Totternhoe Quarry Reserve in 2016 (87 plants) and 2020 (23 plants). Of these, only two plants were Primroses (*P. vulgaris*), with the other 108 being Cowslips (*P. veris*). Larval damaged foodplants were significantly larger than control plants (Table 2A; Fig. 1A), and damaged foodplants were surrounded by more *Primula* plants than undamaged controls (Table 2A; Fig. 1B). Damaged foodplants also had longer leaves than control plants (Table 2A; Fig. 1C). However, there was no evidence of a significant difference in surrounding vegetation height between foodplants and controls (Table 2A; Fig. 1D). Foodplants were significantly closer to scrub when compared to controls at both the smaller (Table 2A; Fig. 1E) and larger scale (Table 2B; Fig. 2A), with increased significance at the larger scale. Foodplants were also in more sheltered locations than unpaired controls (Table 2B; Fig. 2B). Per foodplant, leaves chosen for oviposition were significantly shorter than the longest leaf recorded (Table 2A; (Fig. 1F).

**Table 2:** Comparison of Duke of Burgundy larval damaged foodplants and undamaged control plants at Totternhoe Quarry Reserve in 2016 and 2020

(A) Paired control plants					
Feature of *Primula* plant or surrounding habitat	Foodplants	Control Plants	*n*	Test statistic	*p*
Plant size cm^2^ (± SE)	150.0 (± 16.3)	72.9 (± 7.7)	110 pairs	*V* = 5016.5	< 0.001***
Number of other *Primula* plants within 30 cm (± SE)	3.3 (± 0.2)	2.1 (± 0.2)	110 pairs	*V* = 2866	< 0.001***
Longest leaf length cm (± SE)	12.1 (± 0.4)	10.1 (± 0.4)	110 pairs	*V* = 4051.5	< 0.001***
Surrounding vegetation height cm (± SE)	8.7 (± 0.5)	7.8 (± 0.5)	110 pairs	*V* = 3114	0.103
Distance to scrub cm (± SE)	66.1 (± 5.4)	83.0 (± 7.1)	110 pairs	*V* = 2089.5	0.004**

Feature of *Primula* plant or surrounding habitat	Egg leaf	Longest leaf	*n*	Test statistic	*p*
Length of egg leaf vs longest leaf cm (± SE)	10.6 (± 0.4)	12.1 (± 0.4)	110 pairs	*V* = 1673	< 0.001***

(B) Unpaired control plants					
Feature of *Primula* plant or surrounding habitat	Foodplants	Control plants	*n*	Test statistic	*p*
Distance to scrub cm (± SE)	66.1 (± 5.4)	271.3 (± 30.6)	220	*W* = 2990.5	< 0.001***
Degree of shelter	–	–	220	*X*^*2*^ = 37.3 (*df* = 4)	< 0.001***

For foodplants only, another analysis was carried out comparing the length of their ‘longest leaf’ with that of their ‘egg leaf’, used by Duke of Burgundy for oviposition. Control plants were paired and within one metre of the larval damaged foodplants for all measures (A), apart from the final two listed in the table (B), where control plants were randomly distributed across the reserve to assess differences at a larger scale

**p < 0.01, ***p < 0.001

### Foodplant microclimate

**Fig. 3:**
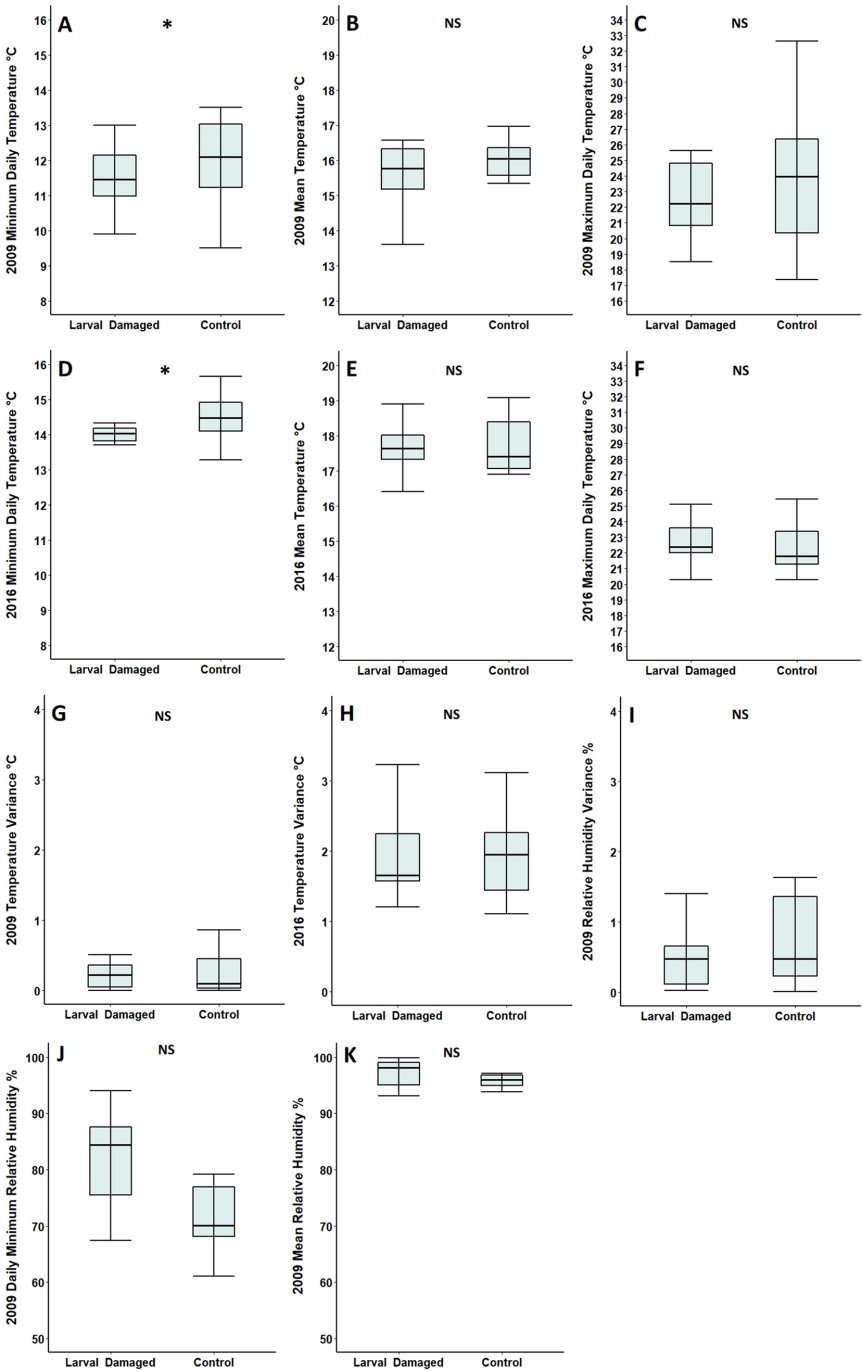
Mean minimum daily temperature, mean temperature and mean maximum daily temperature in **A**, **B**, **C** 2009 and **D**, **E**, **F** 2016; variance in temperature in (**G**) 2009 and (**H**) 2016**;** variance in humidity in 2009 (**I**) and mean minimum daily relative humidity (**J**) and mean relative humidity in 2009 (**K**) of foodplants and paired, control plants on Totternhoe Quarry Reserve. Mean maximum daily humidity was not plotted as all plants recorded a value of 100%. Measurements were taken over the course of two days in 2009 and over the course of one month in 2016. Box and whisker plots show median values, with boxes representing the interquartile range and whiskers extending to the largest value no more than 1.5 × the interquartile range. For ease of readability, outlying data points beyond this range are not displayed. *p < 0.05, NS = no significant difference

In 2009 and 2016, the minimum daily temperatures of foodplants were significantly cooler than paired controls (Table 3; Fig. 3A and D), with minimum daily temperatures of foodplants being an average of 0.5 °C cooler in 2009 and 0.6 °C cooler in 2016. However, in 2009, no differences in mean temperature (Table 3; Fig. 3B), maximum daily temperature (Table 3; Fig. 3C), variance in temperature (Table 3; Fig. 3G), minimum daily humidity (Table 3; Fig. 3J), mean humidity (Table 3; Fig. 3K) or variance in humidity (Table 3; Fig. 3I) were detected between foodplants and paired controls; with the same being true for mean temperature (Table 3; Fig. 3E), maximum daily temperature (Table 3; Fig. 3F) and variance in temperature in 2016 (Table 3; Fig. 3H). Maximum daily humidity levels were the same for foodplants and controls in 2009 (100% humidity across all plants) and therefore were not plotted (Table 3).

**Table 3:** Comparison of microclimatic features of foodplants selected for oviposition and undamaged control plants at Totternhoe Quarry Reserve in 2009 and 2016

Feature of microclimate	Foodplant	Control plant	*n*	Test statistic	*p*
2009					
Minimum daily temperature, °C (± SE)	11.5 (± 0.2)	12.0 (± 0.3)	15 pairs	*V* = 23	0.035*
Mean temperature, °C (± SE)	15.4 (± 0.3)	15.7 (± 0.4)	15 pairs	*V* = 43	0.359
Maximum daily temperature, °C (± SE)	23.0 (± 0.9)	23.7 (± 1.1)	15 pairs	*V* = 46	0.454
Temperature variance (± SE)	0.3 (± 0.1)	0.3 (± 0.1)	15 pairs	*V* = 57	0.890
Minimum daily humidity, % (± SE)	82.0 (± 4.1)	74.0 (± 5.0)	6 pairs	*V* = 15	0.438
Mean humidity, % (± SE)	97.0 (± 1.2)	96.0 (± 0.9)	6 pairs	*V* = 14	0.563
Maximum daily humidity, % (± SE)	100.0 (± 0.0)	100.0 (± 0.0)	–	*–*	–
Humidity variance (± SE)	0.5 (± 0.2)	1.2 (± 0.7)	6 pairs	*V* = 6	0.438
2016					
Minimum daily temperature, °C (± SE)	13.9 (± 0.1)	14.5 (± 0.2)	14 pairs	*V* = 15	0.017*
Mean temperature, °C (± SE)	17.7 (± 0.2)	17.7 (± 0.2)	14 pairs	*V* = 58	0.761
Maximum daily temperature, °C (± SE)	22.5 (± 0.4)	22.3 (± 0.4)	14 pairs	*V* = 58	0.761
Temperature variance (± SE)	2.0 (± 0.2)	2.1 (± 02)	14 pairs	*V* = 57	0.807

Control plants were paired and within one metre of foodplants for all measured features. *p < 0.05

### Cross year comparisons

**Fig. 4:**
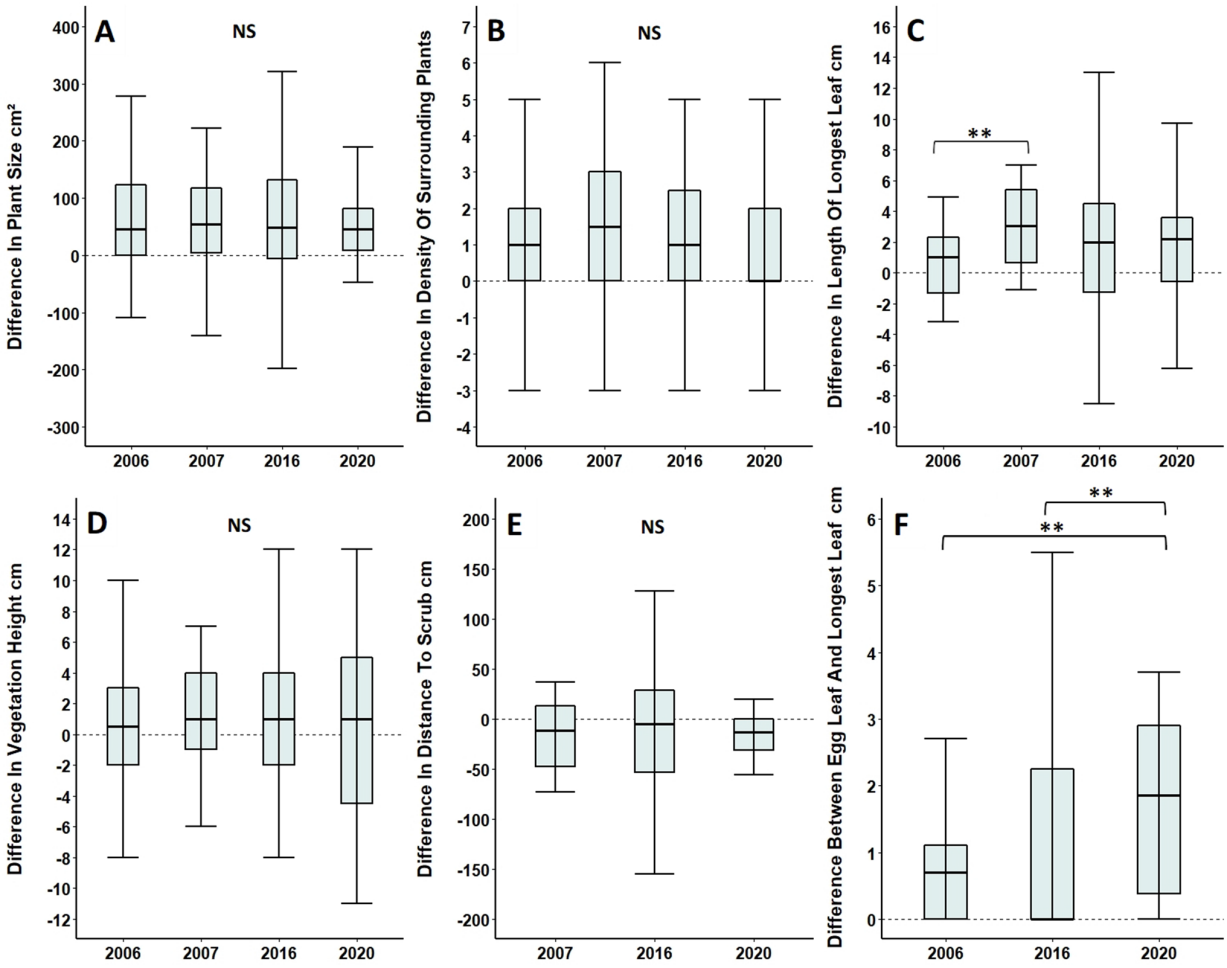
Comparison of differences in** A** plant size, **B** density of surrounding *Primula* plants, **C** longest leaf length, **D** surrounding vegetation height and **E** distance to scrub between foodplants and controls, located at Totternhoe Quarry Reserve in 2006, 2007, 2016 and 2020. All control plants were paired and lie within one metre of a foodplant. **F** Comparison of ‘egg leaf’ length to ‘longest leaf’ length for individual foodplants. Data for distance to scrub and length of egg leaves were not collected for 2006 and 2007 respectively. For **A**–**E**, data falling above the zero line shows that values were greater for foodplants selected for oviposition relative to their controls. For F, data falling above the zero line shows that values were greater for longest leaves than egg leaves. Box and whisker plots show median values, with boxes representing the interquartile range and whiskers extending to the largest value no more than 1.5 × the interquartile range. For ease of readability, outlying data points beyond this range are not displayed. **p < 0.01, NS = no significant difference

Differences between foodplants selected for oviposition and their controls remained consistent across all recorded years (2006, 2007, 2016 and 2020) for both plant size (Table 4; Fig. 4A) and density of surrounding *Primula* plants (Table 4; Fig. 4B), although there was a significant difference found in the length of longest leaf (Table 4; Fig. 4C). Pairwise comparisons using a Dunn’s post hoc test showed that the only significant difference was between 2006 and 2007 (*p* = 0.004), with foodplants in 2006 having relatively shorter leaves when compared to their controls than those in 2007. However, all years followed the same overall trend, with leaves being longer on foodplants selected for oviposition than their controls.

**Table 4:** Cross year comparison of differences between foodplants selected for oviposition and undamaged control plants at Totternhoe Quarry Reserve

Physical feature of *Primula* plant or surrounding habitat	*n*	Test statistic	*p*
Plant size	197	*X*^*2*^ = 0.0566 (*df* = 3)	0.977
Density of surrounding *Primula* plants	197	*X*^*2*^ = 1.94 (*df* = 3)	0.585
Length of longest leaf	197	*X*^*2*^ = 11.4 (*df* = 3)	0.010*
Surrounding vegetation height	197	*X*^*2*^ = 0.0526 (*df* = 3)	0.997
Distance to scrub	146	*X*^*2*^ = 1.55 (*df* = 2)	0.461
Length of egg leaf vs longest leaf	171	*X*^*2*^ = 10.6 (*df* = 2)	0.007**

Feature of microclimate	*n*	Test statistic	*p*
Minimum daily temperature	29	*W* = 98	0.780
Maximum daily temperature	29	*W* = 120	0.533
Mean temperature	29	*W* = 127.5	0.337
Temperature variance	29	*W* = 106	0.983

Measures relating to physical features of *Primula* plants and their surrounding habitat were collected in 2006, 2007, 2016 and 2020, apart from distance to scrub and length of egg leaf, which were not collected for 2006 and 2007 respectively. Microclimatic data was collected in 2009 and 2016. All control plants were paired and lie within one metre of a foodplant

*p < 0.05, **p < 0.01

Differences between foodplants selected for oviposition and their controls also remained consistent across all years of study for surrounding vegetation height (Table 4; Fig. 4D), with the same being true for distance to scrub in 2007, 2016 and 2020 (Table 4; Fig. 4E). However, there was a significant difference between the relative length of egg leaves and longest leaves in 2006, 2016 and 2020 (Table 4) (Fig. 4F). Pairwise comparisons using a Dunn’s post hoc test showed that significant differences occurred between 2020 and 2006 (*p* = 0.006), as well as 2020 and 2016 (*p* = 0.004), with egg leaves in 2020 being even smaller than longest available leaves relative to other years. Again, despite differences in magnitude, all years still followed the same overall trend, with egg leaves tending to be smaller than the longest leaves available on foodplants for oviposition.

**Fig. 5:**
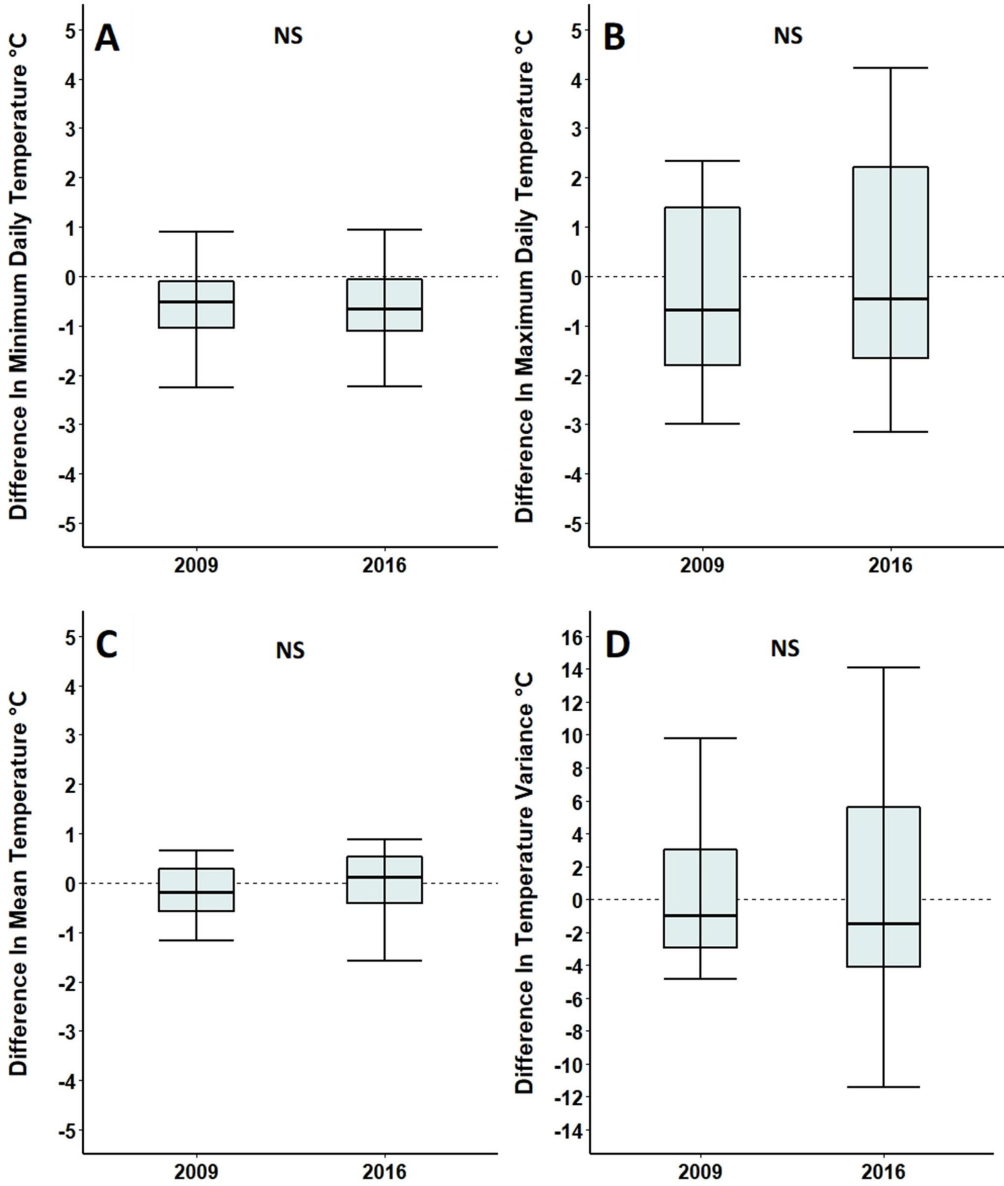
Comparison of differences between larval damaged foodplants and controls, in **A** minimum daily temperature, **B** maximum daily temperature, **C** mean temperature and **D** temperature variance across 2009 and 2016. Measurements were taken over the course of two days in 2009 and one month in 2016. Data falling above the zero line shows that values were greater for foodplants selected for oviposition relative to their controls and vice versa. Box and whisker plots show median values, with boxes representing the interquartile range and whiskers extending to the largest value no more than 1.5 × the interquartile range. For ease of readability, outlying data points beyond this range are not displayed. NS = no significant difference

Differences between foodplants and controls remained consistent for all temperature variables between 2009 and 2016; including minimum daily temperature (Table 4; Fig. 5A), maximum daily temperature (Table 4; Fig. 5B), mean temperature (Table 4; Fig. 5C) and variance in temperature (Table 4; Fig. 5D).

### Scrub clearance and *Primula* regrowth

**Fig. 6:**
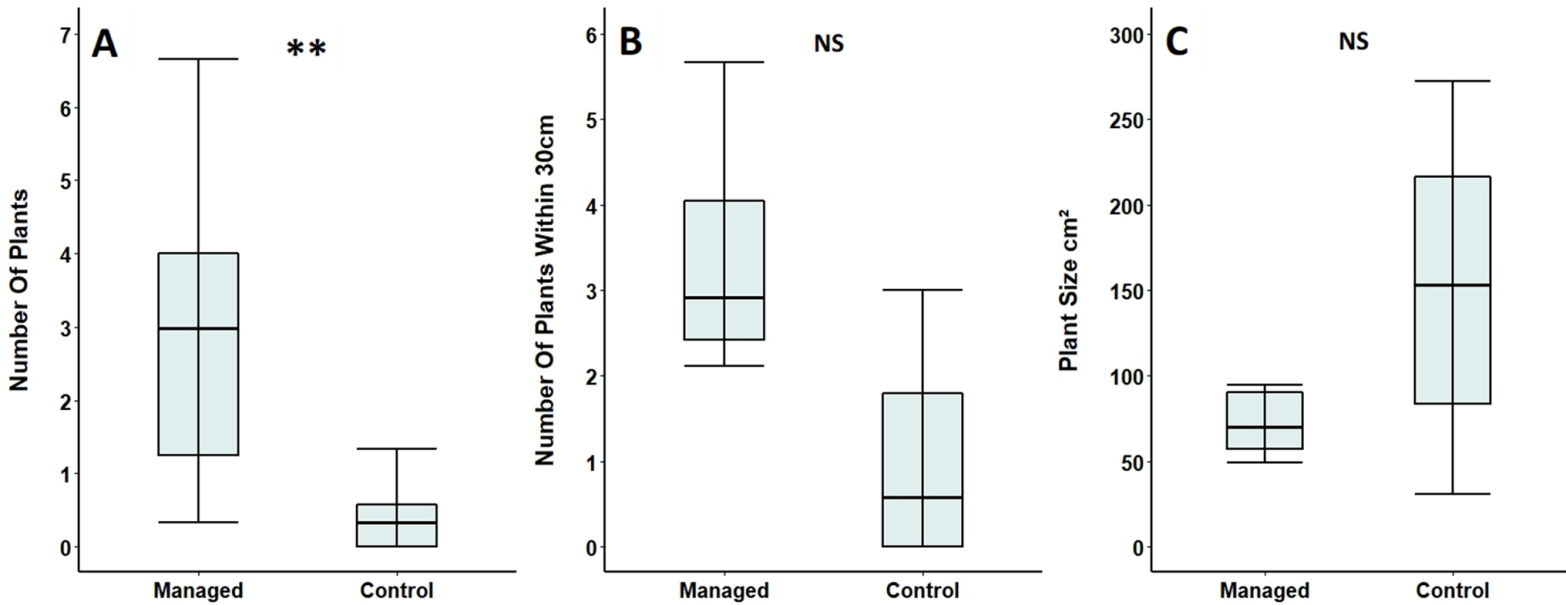
Comparison of the **A** number of *Primula* plants, **B** number of other *Primula* within 30 cm of focal plants and **C** size of *Primula* plants (based on averages from the three sample locations in each plot) in scrub-cleared areas and adjacent uncleared control areas at Totternhoe Quarry Reserve in 2017. If sample areas and their controls did not contain at least one plant each, they were excluded from **B** and **C**. Box and whisker plots are displayed showing median values, with boxes representing the interquartile range and whiskers extending to the largest value no more than 1.5 × the interquartile range. For ease of readability, outlying data points beyond this range are not displayed. **p < 0.01, NS = no significant difference

Scrub-cleared areas had significantly higher numbers of *Primula* plants than adjacent unmanaged control areas, per one-metre diameter sample plots (Table 5; Fig. 6A). However, numbers varied widely, with some sample locations containing no plants, whilst others contained as many as 17 plants. Comparing areas that contained at least one *Primula* plant, we found that the number of *Primula* within 30 cm of focal plants did not differ significantly between cleared and control areas, although there was a trend for managed locations to have a higher density of plants (Table 5; Fig. 6B). The size of *Primula* plants did not differ significantly between managed and control locations (Table 5; Fig. 6C).

**Table 5:** Comparison of *Primula* plants in scrub-cleared areas managed for the Duke of Burgundy and adjacent uncleared control areas (based on averages from three sample locations in each plot) at Totternhoe Quarry Reserve in 2017

Feature of *Primula* plant or surrounding habitat	Managed area	Control area	*n*	Test statistic	*p*
Number of *Primula* plants in area (± SE)	3.0 (± 0.6)	0.4 (± 0.1)	10 pairs	*V* = 55	0.006**
Number of *Primula* within 30 cm of focal plants (± SE)	2.6 (± 0.5)	1.0 (± 0.5)	6 pairs	*V* = 19	0.093
Size of focal *Primula* plants cm^2^ (± SE)	111.0 (± 23.0)	151.2 (± 38.1)	6 pairs	*V* = 5	0.313

If sample locations and their controls did not contain at least one plant each, they were not used in analyses that examined focal plants in more depth

**p < 0.01

**Fig. 7:**
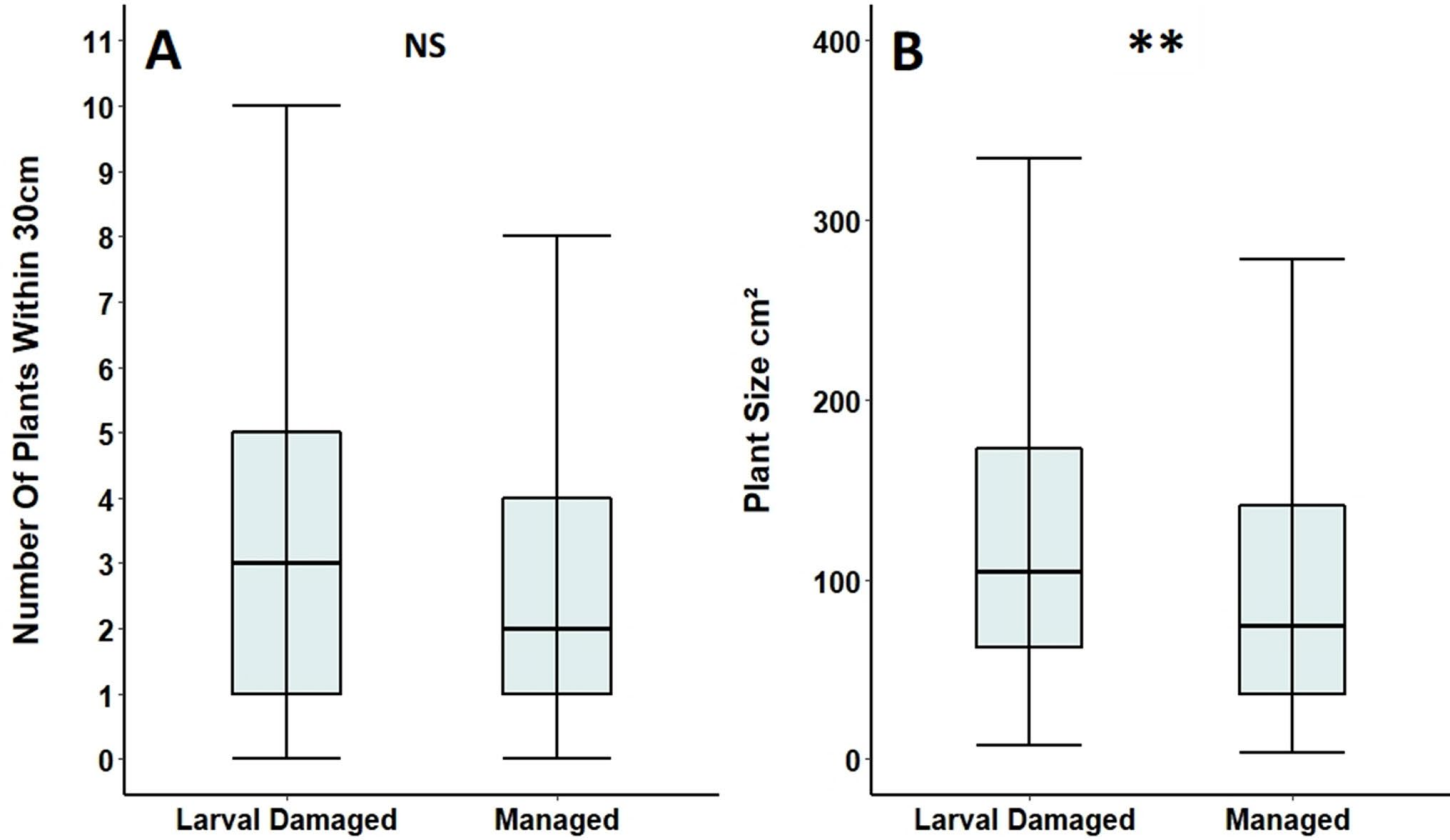
Comparison of **A** the number of *Primula* within 30 cm of larval damaged foodplants and **B** focal plant size in 2016, with undamaged *Primula* plants found in scrub-cleared areas managed at Totternhoe Quarry Reserve in 2017. Box and whisker plots are displayed showing median values, with boxes representing the interquartile range and whiskers extending to the largest value no more than 1.5 × the interquartile range. For ease of readability, outlying data points beyond this range are not displayed. **p < 0.01, NS = no significant difference

Foodplants selected for oviposition in 2016 and *Primula* plants in managed areas in 2017 did not differ significantly in the number of other *Primula* plants found within 30 cm of them (Table 6; Fig. 7A). However, foodplants selected for oviposition were significantly smaller than plants in managed areas (Table 6; Fig. 7B).

**Table 6:** Comparison of Duke of Burgundy larval damaged foodplants found across Totternhoe Quarry Reserve in 2016 and undamaged control plants from scrub-cleared areas managed for the Duke of Burgundy, surveyed in 2017

Feature of *Primula* plant or surrounding habitat	Foodplant	Managed area control plant	*n*	Test statistic	*p*
Number of other *Primula* within 30 cm (± SE)	3.1 (± 0.3)	3.0 (± 0.6)	127	*W* = 1535	0.142
Plant size cm^2^ (± SE)	158.8 (± 19.2)	111.0 (± 23.0)	134	*W* = 1485	0.005**

**p < 0.01

## Discussion

### Key findings

We found that foodplants used by Duke of Burgundy for oviposition differed in their characteristics from other plants, confirming findings of previous studies (Sparks et al. 1994; Fartmann 2006; Anthes et al. 2008; Turner et al. 2009). Both physical and microclimatic features selected for oviposition by the Duke of Burgundy remained consistent across years, highlighting highly conservative foodplant choice by this species. Scrub clearance management at Totternhoe Quarry Reserve promoted some, but not all of the features required for foodplants by this species.

### Foodplant habitat attributes

The largest *Primula* plants in the densest patches were selected for oviposition, providing a large food source. This is likely to benefit larval growth and survival, especially as larvae are known to be able to move short distances to surrounding plants in search of food (Sparks et al. 1994; Oates 2000; Turner et al. 2009). The selected foodplants also had longer leaves than their undamaged, paired controls, but the leaf specifically used for oviposition was often not the largest on the plant. Increased leaf length would supply more food to larvae and is linked to increased overall plant size. However, due to leaves on plants overlapping and larvae being able to travel short distances (Oates 2000; Turner et al. 2009), there would likely be very little benefit in selecting the longest leaf for oviposition, once a suitable plant was found. Our findings suggest that leaf length is not a cue females use to select specific oviposition location on a plant.

Vegetation height did not differ significantly between foodplant and control plant locations and was slightly shorter than the 10–20 cm height generally found in other studies (Butterflies Under Threat Team 1986). This matches findings recorded by Turner et al. (2009) from the same site and could be due to the high overall scrub cover and sheltered slopes at Totternhoe Quarry Reserve (Hayes et al. 2018), meaning microclimates are sufficiently cool for the larvae even in shorter vegetation (Sparks et al. 1994; Kirtley 1995). This is backed up by the finding that foodplants were closer to woody vegetation than controls. Fartmann (2006) found foodplants selected for oviposition could be further from scrub in cooler, wetter environments, as additional shelter was not essential. Taken together, these results indicate that different combinations of factors can provide the same habitat characteristics required by Duke of Burgundy, optimising the speed of egg and larval development, whilst reducing the risk of desiccation (Anthes et al. 2008). Therefore, on the highly sheltered Totternhoe Quarry Reserve, vegetation height may not be as important for determining suitable microclimates for Duke of Burgundy, as it can be in other less-sheltered locations. Another possible contributing factor is that taller surrounding vegetation, while helping to retain moisture, may also hinder a female butterfly’s ability to find or access a *Primula* plant (Anthes et al. 2008), reducing oviposition on plants in taller vegetation.

When control plant locations were randomly selected from across the reserve, significant differences were found between foodplants and controls in characteristics that are likely to influence microclimate. Foodplants selected for oviposition were in more sheltered locations and closer to scrub. Again, this is likely to mean that the foodplants were in cooler and damper locations, benefitting larval growth and survival. However, plants in the highest shelter category were less commonly occupied by larvae than plants in areas with intermediate shelter values. This may be due to similar issues related to plant accessibility in areas with higher vegetation height (Sparks et al. 1994; Anthes et al. 2008), with plants in the most sheltered locations being more difficult for females to find or access. In line with previous findings (Hayes et al. 2018, 2019), it could also be that adult Duke of Burgundy, which require high temperatures to warm up, avoid the most overgrown areas for oviposition, which may be too shaded and cool.

### Foodplant microclimate

Nearly all measures showed no significant difference between foodplants and paired controls. This is surprising, as we expected butterflies to select foodplants that would be less likely to desiccate and these would be likely to have mean or maximum daily temperatures lower and relative humidity higher than controls (Thomas and Simcox 2005; Woon et al. 2019). However, in both years minimum daily temperature of foodplants was significantly cooler. Such lower minimum temperatures at night are difficult to explain, as foodplants were closer to scrub, in areas that should be buffered against the coldest nightly temperatures (Bladon et al. 2020; Kumar et al. 2020). As temperature loggers were placed at the base and centre of *Primula* plants, this finding may reflect differences in ground cover by the foodplants themselves, with larger plants keeping the area more open and exposing their centres to higher cooling at night.

The lack of difference between other aspects of foodplant and control microclimate indicates, at first glance, that microclimate is not as important a factor in determining choice of foodplant as other studies suggest (Sparks et al. 1994; Fartmann 2006; Anthes et al. 2008; Turner et al. 2009). The finding also conflicts with other results from this study, where we propose that proximity to scrub and a high degree of shelter are chosen by females, because they are important for maintaining a suitable microclimate. One explanation for the similarity of microclimatic readings is that all of the control plants were within one metre of foodplants. At this distance, vegetation height, degree of shelter, and consequently microclimatic variables, are likely to vary little, even if microclimate is an important factor for the butterflies in selecting foodplants at larger scales. Having already selected a suitable vegetation structure and microclimate, oviposition choice by female Duke of Burgundy within these areas may, therefore, be more heavily based on plant size and density.

### Cross year comparisons

In spite of variability in temperature and rainfall across years in this study, comparison of foodplants and their controls showed that ovipositional preferences were remarkably consistent between years. Larger plants in denser patches were consistently selected for oviposition and, in all years, selected foodplants had longer leaves than controls, although the longest leaf was not always used as the specific location for oviposition. However, despite trends being conserved across years, some small differences were detected. In 2006, the difference in the lengths of longest leaves between foodplants and controls was significantly smaller than in 2007. In 2020, egg leaves were significantly smaller relative to longest leaves than in 2006 and 2016. These differences are difficult to explain, but may reflect changes in *Primula* growth form between years, related to differing temperature and rainfall, effecting the variability of leaf lengths within and between plants. The variability of egg laying choice in relation to these factors between years provides further support for our finding that leaf length is not as important a feature for oviposition selection as other factors, and perhaps explains why this variable alone varies between years.

Measures of surrounding vegetation height were consistent across years, with no significant difference between foodplants and controls. This provides support for our finding that this feature is not important for Duke of Burgundy oviposition choice, at least at the one metre scale of this study. Distance to scrub appears to be more important as, even when measured at a one metre scale, foodplants were significantly closer to scrub than controls and results were consistent across years. To investigate this further, temperature, humidity and vegetation height could be compared between foodplants and unpaired control plant locations, to assess whether ovipositional preferences in relation to microclimate can be detected at larger scales.

### Current management at Totternhoe Quarry and its effect on Duke of Burgundy

We detected an increase in *Primula* plant abundance in managed areas compared to controls, suggesting that scrub cutting successfully increases the number of plants available to Duke of Burgundy for oviposition on the reserve. Turner et al. (2009) similarly found that more disturbed locations on this site, including steep cliffs, had higher *Primula* abundance. This is probably due to cowslips (*Primula veris*) relying on open ground to germinate successfully and avoid being outcompeted by other plants (Brys et al. 2004). This finding supports the use of regular scrub clearance as an important management tool for supporting high densities of potential foodplants. Cleared areas also had generally higher densities of *Primula* around focal plants compared to control locations, potentially providing a greater food source for larvae, although this difference was not significant. However, cleared areas did not differ significantly from control areas in the size of the plants present, and tended to have smaller *Primula* plants than control sites. When comparing *Primula* plants in cleared areas to chosen larval damaged foodplants in 2016, we found no significant difference in the density of surrounding *Primula,* although plants were significantly smaller in cleared areas. Therefore, although scrub clearance does promote a higher density of *Primula* plants, it does not produce plants in optimal condition for Duke of Burgundy oviposition, as has been suggested by other studies (Turner et al. 2009; Goodenough and Sharp 2016).

Within a given cleared area, there was also a large amount of variation in the number and density of *Primula* plants. The largest difference we found between one metre plots in the same management area was 17 plants, with the plots containing zero and 17 *Primula* plants respectively. This patchy distribution is probably linked to the distribution of long-lived *Primula* seed banks, which can quickly re-emerge when overshadowing vegetation is removed, and may reflect the distribution of *Primula* plants before they were overgrown (Endels et al. 2005).

### Implications for management

Optimal conditions for Duke of Burgundy oviposition consist of large *Primula* plants in dense patches, which are associated with nearby scrub or other shelter. This study has shown that these preferences are remarkably consistent across years, despite inter-annual variation in rainfall, temperature, and number and emergence date of adults. This again highlights the importance of specific management to produce foodplants in the appropriate condition for this selective butterfly and indicates that requirements are unlikely to alter as regional climates change.

Our results support the findings of previous studies and suggest that management for the Duke of Burgundy needs to strike a careful balance between scrub clearance and regrowth (Sparks et al. 1994; Bourn and Warren 1998; Oates 2000; Fartmann 2006; Anthes et al. 2008; Turner et al. 2009; Hayes et al. 2018). Some form of periodic clearance is needed to maintain short vegetation and a high abundance of *Primula* plants, but enough time needs to elapse between management to allow plants to reach optimal condition for oviposition (Goodenough and Sharp 2016). 90% of the areas managed for the Duke of Burgundy analysed in this study had been cleared within the last 18 months and were still very exposed, perhaps explaining why plants were generally still small in these locations. As others have suggested, a long-term rotation, clearing blocks of scrub on grassland sites every 2–4 years, could offer a solution (Warren and Thomas 1992); allowing moderately sized, sheltered *Primula* plants to grow up before being swamped by surrounding vegetation (Sparks et al. 1994; Goodenough and Sharp 2016). However, the correct time frame for management needs to be coupled with the correct scale of scrub clearance and, even then, the benefits in terms of plant recolonization are likely to be variable. Targeting clearance to the edges of dense scrub blocks, where existing large *Primula* plants are yet to be shaded out, may restore areas known to contain *Primula* seed banks, whilst reducing wasted management effort (Endels et al. 2005). This approach should also reduce the number of *Primula* plants exposed in the centre of large cleared areas and at risk of dessication (Sparks et al. 1994; Goodenough and Sharp 2016). The timing and intensity of clearance will need to be refined, following more work analysing its joint effects on *Primula* plant abundance, size and condition (Goodenough and Sharp 2016), and how long it takes the Duke of Burgundy to colonise and lay eggs in newly managed areas (Sparks et al. 1994).

Continued monitoring to assess whether Duke of Burgundy move in and use cleared areas will be essential to the success of any management programme. Where possible, combining scrub clearance with moderate intensity autumn cattle grazing may help to better maintain a continuity of transitional habitat required to support the Duke of Burgundy (Sparks et al. 1994; Bourn and Warren 1998; Fartmann 2006; Goodenough and Sharp 2016). Maintaining an early successional stage with this form of management should also support other species of butterfly that are of conservation concern and help propagate their foodplants (Krämer et al. 2012). Furthermore, benefits should extend beyond butterflies to a wide range of taxa. Breaking up areas of homogenous scrub and expanding warm fringe habitat has been shown to enhance the conservation value of calcareous grasslands by increasing habitat quality and heterogeneity, supporting numerous target species (Poniatowski et al. 2020). Implementing management at a landscape-scale and linking up small isolated sites like Totternhoe Quarry would allow successional habitat to be supported across a larger region, and reduce the need to maintain ‘permanent’ transitional habitats on individual reserves (Hayes et al. 2019).

## Conclusion

As with previous studies, we found evidence that the Duke of Burgundy has very specific habitat requirements and that eggs are only laid on a small subset of available plants. Despite interannual variability in temperature and rainfall, ovipositional preferences were found to be consistent over a 14-year timeframe. Current management consisting of manual scrub clearance maintains some but not all of these preferences and more work is needed to identify management options that can continue to maintain suitable habitat conditions for the Duke of Burgundy on transitional sites such as Totternhoe Quarry.

Beyond butterflies, identifying consistencies in preferences across years will be important for a wide range of species and more multiyear studies are needed to help inform long term management plans. Whether preferences are extremely consistent, as for the Duke of Burgundy, or more variable from year to year, maintaining suitable conditions will be made more feasible by increasing heterogeneity on reserves. As climates continue to change, management plans that promote habitat and topographic variability are set to become increasingly important.

## Declarations

### Research involving animals

No butterflies were harmed during the course of this project. All work was carried out with the full consent of the BCN Wildlife Trust on one of their reserves and they work closely with Natural England to ensure the best monitoring of species on their sites. Natural England also provided the BCN Wildlife Trust with written consent to undertake surveys. Please see ‘Consent of Natural England’ form.

## References

[CR1] Anthes N, Fartmann T, Hermann G (2008). The Duke of Burgundy butterfly and its dukedom: larval niche variation in *Hamearis lucina* across Central Europe. J Insect Conserv.

[CR2] ArcMap (2020) https://desktop.arcgis.com/en/arcmap/ (accessed 19 Oct 2020)

[CR3] Awmack CS, Leather SR (2002). Host plant quality and fecundity in herbivorous insects. Annu Rev Entomol.

[CR4] BCN Wildlife Trust, Totternhoe (2020) https://www.wildlifebcn.org/nature-reserves/totternhoe (accessed 19 Oct 2020)

[CR5] Beyer LJ, Schultz CB (2010). Oviposition selection by a rare grass skipper *Polites mardon* in montane habitats: Advancing ecological understanding to develop conservation strategies. Biol Conserv.

[CR6] Bladon AJ, Lewis M, Bladon EK, Buckton SJ, Corbett S, Ewing SR, Hayes MP, Hitchcock GE, Knock R, Lucas C, McVeigh A, Menéndez R, Walker JM, Fayle TM, Turner EC (2020). How butterflies keep their cool: physical and ecological traits influence thermoregulatory ability and population trends. J Anim Ecol.

[CR7] Bourn NAD & Warren MS (1998) *Species Action Plan: Duke of Burgundy Hamearis lucina*. Butterfly Conservation, Wareham, Dorset.

[CR8] Brys R, Jacquemyn H, Endels P, De Blust G, Hermy M (2004). The effects of grassland management on plant performance and demography in the perennial herb *Primula veris*. J Appl Ecol.

[CR9] Butterflies Under Threat Team (1986) *The management of chalk grassland for butterflies*. *Focus on nature conservation No 17.* Nature Conservancy Council, Peterborough.

[CR10] Chew FS (1977). Coevolution of pierid butterflies and their cruciferous food plants. II. The distribution of eggs on potential food plants. Evolution.

[CR11] Dennis RLH, Hodgson JG, Grenyer R, Shreeve TG, Roy DB (2004). Host plants and butterfly biology. Do host-plant strategies drive butterfly status?. Ecol Entomol.

[CR12] Dennis RLH, Shreeve T (1991). Climatic change and the British butterfly fauna: opportunities and constraints. Biol Conserv.

[CR13] Dennis RLH, Shreeve TG, Arnold HR, Roy DB (2005). Does diet breadth control herbivorous insect distribution size? Life history and resource outlets for specialist butterflies. J Insect Conserv.

[CR14] Elmes GW, Wardlaw JC (1982). A population study of the ants *Myrmica sabuleti* and *Myrmica scabrinodis*, living at two sites in the south of England. I. A comparison of colony populations. J Anim Ecol.

[CR15] Elmes GW, Wardlaw JC (1982). A population study of the ants *Myrmica sabuleti* and *Myrmica scabrinodis*, living at two sites in the south of England. II. Effects of above-nest vegetation. J Anim Ecol.

[CR16] Endels P, Jacquemyn H, Brys R, Hermy M (2005). Rapid response to habitat restoration by the perennial *Primula veris* as revealed by demographic monitoring. Plant Ecol.

[CR17] Fartmann T (2006). Oviposition preferences, adjacency of old woodland and isolation explain the distribition of the Duke of Burgundy butterfly (*Hamearis lucina*) in calcareous grasslands in central Germany. Ann Zool Fenn.

[CR19] García-Barros E, Fartmann T, Settele J, Shreeve TG, Konvička M, van Dyck H (2009). Butterfly oviposition: sites, behaviour and modes. Ecology of butterflies in Europe.

[CR20] Goldenberg SE (2004) Influence of foodplant selection on the successful development of caterpillars of the Duke of Burgundy butterfly. MSc Thesis. Cranfield University, Silsoe, UK

[CR21] Goodenough AE, Sharp M (2016). Managing calcareous grassland for the declining Duke of Burgundy Hamearis lucina butterfly: effects of grazing management on Primula host plants. J Insect Conserv.

[CR22] Hayes MP, Hitchcock GE, Knock RI, Lucas CBH, Turner EC (2019). Temperature and territoriality in the Duke of Burgundy butterfly, *Hamearis Lucina*. J Insect Conserv.

[CR23] Hayes MP, Rhodes MW, Hitchcock GE, Knock RI, Lucas CBH, Chaney PK, Turner EC (2018). Determining the long-term habitat preferences of the Duke of Burgundy butterfly, *Hamearis lucina*, on a chalk grassland reserve in the UK. J Insect Conserv.

[CR24] Intergovernmental Panel on Climate Change (2014) Climate Change 2014: Synthesis Report. Contribution of Working Groups I, II and III to the Fifth Assessment Report of the Intergovernmental Panel on Climate Change. Intergovernmental Panel on Climate Change, Geneva, Switzerland

[CR25] ImageJ (2020) https://imagej.net/ImageJ (accessed 19 Oct 2020)

[CR26] Jones R, Ellis S, Hoare D, Wainwright D & Rosenthal A (2012) *Status and conservation of the Duke of the Burgundy Hamearis lucina butterfly in England*. Butterfly Conservation, Dorset Report no. S13–19

[CR27] Kirtley S (1995) The current status and ecology of the Duke of Burgundy butterfly (*Hamearis lucina* L.) in South Cumbria and North Lancashire. Report to English Nature

[CR28] Kirtley S (1997) The current status and ecology of the Duke of Burgundy butterfly (*Hamearis lucina* L.) in South Cumbria and North Lancashire. Report to English Nature

[CR29] Krämer B, Kämpf I, Enderle J, Poniatowski D, Fartmann T (2012). Microhabitat selection in a grassland butterfly: a trade-off between microclimate and food availability. J Insect Conserv.

[CR30] Kumar SS, Prihodko L, Lind BM, Anchang J, Ji W, Ross CW, Kahiu MN, Velpuri NM, Hanan NP (2020). Remotely sensed thermal decay rate: an index for vegetation monitoring. Sci Rep.

[CR31] Kurze S, Heinken T, Fartmann T (2018). Nitrogen enrichment in host plants increases the mortality of common Lepidoptera species. Oecologia.

[CR32] Leon-Cortes JL, Perez-Espinoza F, Marin L, Molina-Martinez A (2004). Complex habitat requirements and conservation needs of the only extant Baroniinae swallowtail butterfly. Anim Conserv.

[CR33] Loffler F, Stuhldreher G, Fartmann T (2013). How much care does a shrub-feeding hairstreak butterfly, *Satyrium spini* (Lepidoptera: Lycaenidae), need in calcareous grasslands?. Eur J Entomol.

[CR34] Met Office (2006) MIDAS: UK daily temperature data. NCAS British Atmospheric Data Centre. https://catalogue.ceda.ac.uk/uuid/1bb479d3b1e38c339adb9c82c15579d8 (accessed 2 Oct 2020)

[CR35] Nakonieczny M, Kedziorski A (2005). Feeding preferences of the Apollo butterfly (*Parnassius apollo* ssp. frankenbergeri) larvae inhabiting the Pieniny Mts (southern Poland). C R Biol.

[CR36] Newman E (1871). An illustrated natural history of British butterflies and moths.

[CR37] Noake B, Bulman C & Bourn N (eds.) (2008) Action for the Duke of Burgundy: sharing good practice. Proceedings from a Butterfly Conservation Seminar. Butterfly Conservation Report S08–33, Wareham, Dorset

[CR39] Oates M (2000). The Duke of Burgundy—conserving the intractable. Br Wildl.

[CR40] Poniatowski D, Stuhldreher G, Helbing F, Hamer U, Fartmann T (2020) Restoration of calcareous grasslands: the early successional stage promotes biodiversity. Ecol Eng 151:105858.

[CR41] Sparks TH, Porter K, Greatorex-Davies JN, Hall ML, Marrs RH (1994). The choice of oviposition sites in woodland by the Duke of Burgundy butterfly *Hamearis lucina* in England. Biol Conserv.

[CR42] Stewart KEJ, Bourn NAD, Thomas JA (2001). An evaluation of three quick methods commonly used to assess sward height in ecology. J Appl Ecol.

[CR43] Thomas JA, Simcox DJ, Settele J, Kuehn E, Thomas JA (2005). Contrasting management requirements of Maculinea arion across latitudinal and altitudinal climatic gradients in west Europe. Studies on the ecology and conservation of butterflies in Europe: species ecology along a European gradient: Maculinea butterflies as a model.

[CR44] Turner EC, Granroth HMV, Johnson HR, Lucas CBH, Thompson AM, Froy H, German RN, Holdgate R (2009). Habitat preference and dispersal of the Duke of Burgundy butterfly (*Hamearis lucina*) on an abandoned chalk quarry in Bedfordshire, UK. J Insect Conserv.

[CR45] UKBMS, Methods (2020) https://www.ukbms.org/Methods (accessed 17 Dec 2020)

[CR46] Warren MS, Thomas JA, Buckley GP (1992). Butterfly responses to coppicing. Ecology and management of coppice woodlands.

[CR47] Woon JS, Boyle MJW, Ewers RM, Chung A, Eggleton P (2019). Termite environmental tolerances are more linked to desiccation than temperature in modified tropical forests. Insect Soc.

